# 4-Aza-1-azoniabicyclo­[2.2.2]octa­ne–2-amino­benzoate–2-amino­benzoic acid (1/1/1)

**DOI:** 10.1107/S1600536811041559

**Published:** 2011-10-12

**Authors:** Hadi D. Arman, Trupta Kaulgud, Edward R. T. Tiekink

**Affiliations:** a Department of Chemistry, The University of Texas at San Antonio, One UTSA Circle, San Antonio, Texas 78249-0698, USA; b Department of Chemistry, University of Malaya, 50603 Kuala Lumpur, Malaysia

## Abstract

A 4-aza-1-azoniabicyclo­[2.2.2]octane cation, a 2-amino­benzoate anion and a neutral 2-amino­benzoic acid mol­ecule comprise the asymmetric unit of the title compound, C_6_H_13_N_2_^+^·C_7_H_6_NO_2_^−^·C_7_H_7_NO_2_. An intra­molecular N—H⋯O hydrogen bond occurs in the anion and in the neutral 2-amino­benzoic acid mol­ecule. The cation provides a charge-assisted N—H⋯O hydrogen bond to the anion, and the 2-amino­benzoic acid mol­ecule forms an O—H⋯N hydrogen bond to the unprotonated amino N atom in the cation. In this way, a three-component aggregate is formed. These are connected into a three-dimensional network by amino–carboxyl­ate N—H⋯O hydrogen bonds. N—H⋯N hydrogen bonds are also observed.

## Related literature

For related studies on co-crystal formation, see: Arman *et al.* (2010 ▶); Arman & Tiekink (2010 ▶); Wardell & Tiekink (2011 ▶). For examples of multi-component crystals containing the 2-amino­benzoate anion, see: Lynch *et al.* (1998 ▶); Chen & Peng (2011 ▶). For a description of the Cambridge Structural Database, see: Allen (2002 ▶).
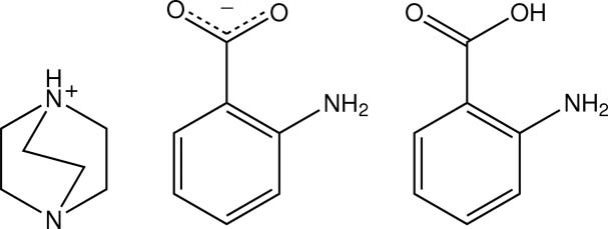


## Experimental

### 

#### Crystal data

C_6_H_13_N_2_^+^·C_7_H_6_NO_2_^−^·C_7_H_7_NO_2_
*M* *_r_* = 386.45
Monoclinic, 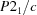

*a* = 9.285 (3) Å
*b* = 16.843 (5) Å
*c* = 12.660 (4) Å
β = 102.127 (6)°
*V* = 1935.7 (10) Å^3^
*Z* = 4
Mo *K*α radiation
μ = 0.09 mm^−1^
*T* = 98 K
0.34 × 0.17 × 0.07 mm

#### Data collection

Rigaku AFC12/SATURN724 diffractometer
Absorption correction: multi-scan (*ABSCOR*; Higashi, 1995 ▶) *T*_min_ = 0.731, *T*_max_ = 1.000
16911 measured reflections
4440 independent reflections
3929 reflections with *I* > 2σ(*I*)
*R*_int_ = 0.054

#### Refinement

*R*[*F* ^2^ > 2σ(*F* ^2^)] = 0.056
*wR*(*F* ^2^) = 0.128
*S* = 1.13
4440 reflections
268 parameters
7 restraints
H-atom parameters constrained
Δρ_max_ = 0.30 e Å^−3^
Δρ_min_ = −0.23 e Å^−3^

### 

Data collection: *CrystalClear* (Molecular Structure Corporation & Rigaku, 2005 ▶); cell refinement: *CrystalClear*; data reduction: *CrystalClear*; program(s) used to solve structure: *SHELXS97* (Sheldrick, 2008 ▶); program(s) used to refine structure: *SHELXL97* (Sheldrick, 2008 ▶); molecular graphics: *ORTEPII* (Johnson, 1976 ▶) and *DIAMOND* (Brandenburg, 2006 ▶); software used to prepare material for publication: *publCIF* (Westrip, 2010 ▶).

## Supplementary Material

Crystal structure: contains datablock(s) global, I. DOI: 10.1107/S1600536811041559/hb6440sup1.cif

Structure factors: contains datablock(s) I. DOI: 10.1107/S1600536811041559/hb6440Isup2.hkl

Supplementary material file. DOI: 10.1107/S1600536811041559/hb6440Isup3.cml

Additional supplementary materials:  crystallographic information; 3D view; checkCIF report

## Figures and Tables

**Table 1:** Hydrogen-bond geometry (Å, °)

*D*—H⋯*A*	*D*—H	H⋯*A*	*D*⋯*A*	*D*—H⋯*A*
O1—H1O⋯N3	0.84	1.77	2.597 (2)	168
N4—H5*n*⋯O3	0.93	1.64	2.546 (2)	166
N1—H2*n*⋯O2	0.88	2.03	2.725 (2)	135
N2—H3*n*⋯O3	0.88	2.04	2.696 (2)	131
N1—H1*n*⋯O4^i^	0.88	2.08	2.941 (2)	165
N2—H4*n*⋯N1^ii^	0.88	2.38	3.256 (2)	171

Symmetry codes: (i) 
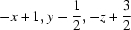
; (ii) 
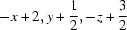
.

**Fig. 1.**
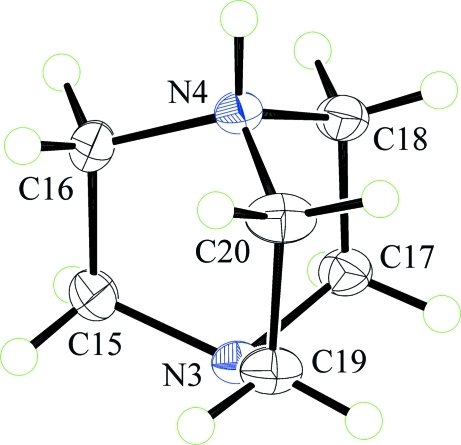
Molecular structure of the 4-aza-1-azoniabicyclo(2.2.2)octane cation in (I) showing atom-labelling scheme and displacement ellipsoids at the 50% probability level.

**Fig. 2.**
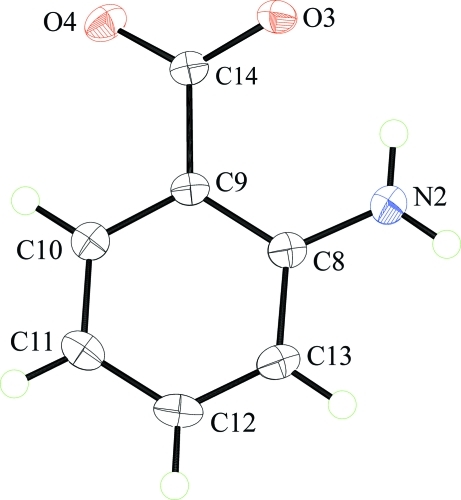
Molecular structure of the 2-aminobenzoate anion in (I) showing atom-labelling scheme and displacement ellipsoids at the 50% probability level.

**Fig. 3.**
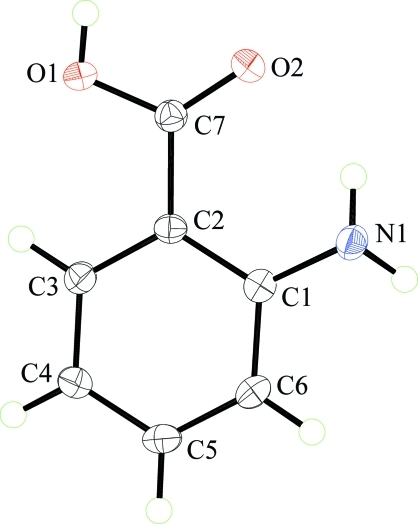
Molecular structure of the neutral 2-aminobenzoic acid molecule in (I) showing atom-labelling scheme and displacement ellipsoids at the 50% probability level.

**Fig. 4.**
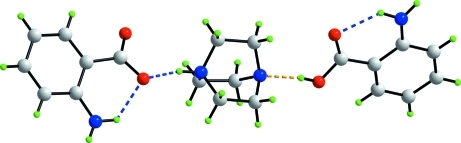
Three component aggregate in (I) held together by O—H···N and N—H···O hydrogen bonds shown as orange and blue dashed lines, respectively.

**Fig. 5.**
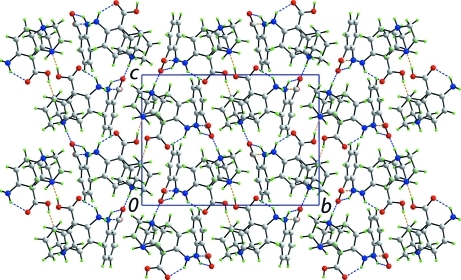
View in projection down the a axis of the unit-cell contents of (I). The O—H···N and N—H···O hydrogen bonds are shown as orange and blue dashed lines, respectively.

## supplementary crystallographic information

### Comment

As a part of on-going studies into co-crystallization experiments of carboxylic acids with N-containing molecules (Arman *et al.* 2010; Arman & Tiekink, 2010; Wardell & Tiekink, 2011), the 1:2 co-crystallization of 1,4-diazabicyclo[2.2.2]octane (DABCO) and 2-aminobenzoic was investigated, leading to the isolation of (I).

The crystallographic asymmetric unit of (I) comprises a 4-aza-1-azoniabicyclo(2.2.2)octane cation, Fig. 1, a 2-aminobenzoate anion, Fig. 2, and a neutral 2-aminobenzoic acid molecule, Fig. 3. While there are many examples of 4-aza-1-azoniabicyclo(2.2.2)octane cations and neutral 2-aminobenzoic acid molecules in the crystallographic literature (Allen, 2002), examples of 2-aminobenzoate anions are comparatively rare in all-organic molecules (Lynch *et al.*, 1998; Chen & Peng, 2011). The ions and neutral benzoic acid derivative associate into a three-molecule aggregate *via* N^+^—H···O and O—H···N hydrogen bonds formed by and to the cation, Fig. 4 and Table 1; intramolecular N—H···O hydrogen bonds are also noted in the benzoate and benzoic acid species, Table 1.

The three component aggregates are connected into the three-dimensional architecture by hydrogen bonds involving the amino-H atoms not participating in intramolecular N—H···O interactions, Fig. 5 and Table 1.

### Experimental

Colourless crystals of (I) were isolated from the 1:2 co-crystallization of 1,4-diazabicyclo[2.2.2]octane (Sigma-Aldrich, 0.089 mmol) and anthranilic acid (Sigma-Aldrich, 0.19 mmol) in chloroform solution (6 ml); *M*.pt. 427–430 K.

### Refinement

The C-bound H-atoms were placed in calculated positions (C—H 0.95–0.99 Å) and were included in the refinement in the riding model approximation with *U*_iso_(H) set to 1.2*U*_eq_(C). The O– and N-bound H-atoms were located in a difference Fourier map and were refined with distance restraints of O—H 0.840±0.001 Å and N—H = 0.088±0.001 Å, respectively, and with *U*_iso_(H) = 1.5*U*_eq_(O, N).

### Crystal data

**Table:**

C_6_H_13_N_2_^+^·C_7_H_6_NO_2_^−^·C_7_H_7_NO_2_	*F*(000) = 824
*M**_r_* = 386.45	*D*_x_ = 1.326 Mg m^−^^3^
Monoclinic, *P*2_1_/*c*	Mo *K*α radiation, λ = 0.71073 Å
Hall symbol: -P 2ybc	Cell parameters from 8162 reflections
*a* = 9.285 (3) Å	θ = 2.0–40.6°
*b* = 16.843 (5) Å	µ = 0.09 mm^−^^1^
*c* = 12.660 (4) Å	*T* = 98 K
β = 102.127 (6)°	Block, colourless
*V* = 1935.7 (10) Å^3^	0.34 × 0.17 × 0.07 mm
*Z* = 4	

### Data collection

**Table:**

Rigaku AFC12K/SATURN724 diffractometer	4440 independent reflections
Radiation source: fine-focus sealed tube	3929 reflections with *I* > 2σ(*I*)
graphite	*R*_int_ = 0.054
ω scans	θ_max_ = 27.5°, θ_min_ = 2.0°
Absorption correction: multi-scan (*ABSCOR*; Higashi, 1995)	*h* = −12→12
*T*_min_ = 0.731, *T*_max_ = 1.000	*k* = −19→21
16911 measured reflections	*l* = −15→16

### Refinement

**Table:**

Refinement on *F*^2^	Primary atom site location: structure-invariant direct methods
Least-squares matrix: full	Secondary atom site location: difference Fourier map
*R*[*F*^2^ > 2σ(*F*^2^)] = 0.056	Hydrogen site location: inferred from neighbouring sites
*wR*(*F*^2^) = 0.128	H-atom parameters constrained
*S* = 1.13	*w* = 1/[σ^2^(*F*_o_^2^) + (0.0473*P*)^2^ + 0.9109*P*] where *P* = (*F*_o_^2^ + 2*F*_c_^2^)/3
4440 reflections	(Δ/σ)_max_ < 0.001
268 parameters	Δρ_max_ = 0.30 e Å^−^^3^
7 restraints	Δρ_min_ = −0.23 e Å^−^^3^

### Special details

**Table:**

Geometry. All e.s.d.'s (except the e.s.d. in the dihedral angle between two l.s. planes) are estimated using the full covariance matrix. The cell e.s.d.'s are taken into account individually in the estimation of e.s.d.'s in distances, angles and torsion angles; correlations between e.s.d.'s in cell parameters are only used when they are defined by crystal symmetry. An approximate (isotropic) treatment of cell e.s.d.'s is used for estimating e.s.d.'s involving l.s. planes.
Refinement. Refinement of *F*^2^ against ALL reflections. The weighted *R*-factor *wR* and goodness of fit *S* are based on *F*^2^, conventional *R*-factors *R* are based on *F*, with *F* set to zero for negative *F*^2^. The threshold expression of *F*^2^ > σ(*F*^2^) is used only for calculating *R*-factors(gt) *etc*. and is not relevant to the choice of reflections for refinement. *R*-factors based on *F*^2^ are statistically about twice as large as those based on *F*, and *R*- factors based on ALL data will be even larger.

### Fractional atomic coordinates and isotropic or equivalent isotropic displacement parameters (Å^2^)

**Table:**

	*x*	*y*	*z*	*U*_iso_*/*U*_eq_	
O1	0.73506 (15)	0.46965 (8)	1.01396 (10)	0.0286 (3)	
H1o	0.7433	0.4752	0.9495	0.043*	
O2	0.63182 (15)	0.35565 (8)	0.94734 (10)	0.0280 (3)	
O3	0.87552 (13)	0.60731 (8)	0.46906 (9)	0.0255 (3)	
O4	0.63909 (13)	0.62403 (7)	0.38987 (10)	0.0245 (3)	
N1	0.58477 (16)	0.24385 (8)	1.09196 (12)	0.0225 (3)	
H1n	0.5262	0.2075	1.1100	0.034*	
H2n	0.5710	0.2596	1.0243	0.034*	
N2	1.07213 (17)	0.69313 (11)	0.38788 (13)	0.0320 (4)	
H3n	1.0547	0.6697	0.4460	0.048*	
H4n	1.1631	0.7060	0.3850	0.048*	
N3	0.76627 (15)	0.50739 (8)	0.82113 (11)	0.0203 (3)	
N4	0.79912 (15)	0.55587 (8)	0.63832 (11)	0.0207 (3)	
H5n	0.8114	0.5735	0.5712	0.025*	
C1	0.61784 (17)	0.30546 (9)	1.16546 (13)	0.0184 (3)	
C2	0.66288 (17)	0.38151 (9)	1.13704 (12)	0.0175 (3)	
C3	0.69563 (17)	0.44053 (10)	1.21593 (13)	0.0200 (3)	
H3	0.7229	0.4918	1.1959	0.024*	
C4	0.68936 (19)	0.42613 (11)	1.32282 (13)	0.0232 (3)	
H4	0.7110	0.4671	1.3754	0.028*	
C5	0.65063 (18)	0.35033 (10)	1.35151 (13)	0.0228 (3)	
H5	0.6493	0.3392	1.4249	0.027*	
C6	0.61417 (17)	0.29115 (10)	1.27466 (13)	0.0209 (3)	
H6	0.5863	0.2402	1.2957	0.025*	
C7	0.67418 (17)	0.40032 (10)	1.02372 (13)	0.0192 (3)	
C8	0.96688 (18)	0.68766 (10)	0.29348 (13)	0.0211 (3)	
C9	0.82171 (17)	0.66015 (9)	0.29093 (13)	0.0185 (3)	
C10	0.72053 (18)	0.65777 (10)	0.19229 (13)	0.0211 (3)	
H10	0.6227	0.6407	0.1912	0.025*	
C11	0.7582 (2)	0.67941 (10)	0.09614 (14)	0.0253 (4)	
H11	0.6883	0.6759	0.0298	0.030*	
C12	0.9008 (2)	0.70645 (10)	0.09858 (14)	0.0259 (4)	
H12	0.9283	0.7215	0.0333	0.031*	
C13	1.00247 (19)	0.71146 (10)	0.19520 (14)	0.0243 (4)	
H13	1.0983	0.7313	0.1955	0.029*	
C14	0.77248 (18)	0.62962 (9)	0.38957 (13)	0.0190 (3)	
C15	0.92485 (19)	0.50551 (11)	0.81698 (14)	0.0254 (4)	
H15A	0.9641	0.4512	0.8332	0.031*	
H15B	0.9806	0.5420	0.8721	0.031*	
C16	0.94441 (19)	0.53077 (12)	0.70394 (15)	0.0289 (4)	
H16A	1.0154	0.5753	0.7103	0.035*	
H16B	0.9837	0.4858	0.6682	0.035*	
C17	0.71221 (19)	0.58987 (10)	0.80251 (14)	0.0241 (4)	
H17A	0.7639	0.6242	0.8620	0.029*	
H17B	0.6055	0.5916	0.8022	0.029*	
C18	0.7387 (2)	0.62134 (10)	0.69428 (14)	0.0262 (4)	
H18A	0.6450	0.6406	0.6491	0.031*	
H18B	0.8091	0.6662	0.7070	0.031*	
C19	0.6829 (2)	0.45573 (11)	0.73500 (13)	0.0255 (4)	
H19A	0.5780	0.4541	0.7403	0.031*	
H19B	0.7224	0.4010	0.7443	0.031*	
C20	0.6952 (2)	0.48755 (11)	0.62315 (14)	0.0294 (4)	
H20A	0.7318	0.4452	0.5813	0.035*	
H20B	0.5972	0.5048	0.5826	0.035*	

### Atomic displacement parameters (Å^2^)

**Table:**

	*U*^11^	*U*^22^	*U*^33^	*U*^12^	*U*^13^	*U*^23^
O1	0.0416 (7)	0.0283 (7)	0.0170 (6)	−0.0134 (5)	0.0084 (5)	0.0005 (5)
O2	0.0388 (7)	0.0268 (7)	0.0190 (6)	−0.0089 (5)	0.0072 (5)	−0.0038 (5)
O3	0.0244 (6)	0.0334 (7)	0.0195 (6)	−0.0001 (5)	0.0065 (5)	0.0052 (5)
O4	0.0217 (6)	0.0242 (6)	0.0305 (7)	0.0010 (5)	0.0116 (5)	0.0023 (5)
N1	0.0269 (7)	0.0176 (7)	0.0247 (7)	−0.0022 (5)	0.0093 (6)	−0.0011 (6)
N2	0.0226 (7)	0.0477 (10)	0.0253 (8)	−0.0078 (7)	0.0038 (6)	0.0046 (7)
N3	0.0226 (7)	0.0212 (7)	0.0176 (6)	−0.0018 (5)	0.0052 (5)	0.0014 (5)
N4	0.0224 (7)	0.0229 (7)	0.0181 (6)	0.0010 (5)	0.0072 (5)	0.0027 (5)
C1	0.0162 (7)	0.0179 (8)	0.0214 (8)	0.0017 (6)	0.0048 (6)	0.0006 (6)
C2	0.0168 (7)	0.0196 (8)	0.0164 (7)	0.0012 (6)	0.0044 (6)	0.0009 (6)
C3	0.0210 (7)	0.0192 (8)	0.0203 (8)	−0.0014 (6)	0.0057 (6)	0.0006 (6)
C4	0.0265 (8)	0.0257 (9)	0.0179 (8)	−0.0038 (6)	0.0055 (6)	−0.0018 (7)
C5	0.0230 (8)	0.0286 (9)	0.0173 (8)	0.0001 (6)	0.0051 (6)	0.0046 (7)
C6	0.0213 (8)	0.0185 (8)	0.0236 (8)	0.0013 (6)	0.0067 (7)	0.0049 (6)
C7	0.0195 (7)	0.0191 (8)	0.0193 (8)	0.0001 (6)	0.0046 (6)	0.0001 (6)
C8	0.0225 (8)	0.0192 (8)	0.0226 (8)	0.0023 (6)	0.0069 (6)	0.0009 (6)
C9	0.0220 (8)	0.0151 (7)	0.0199 (8)	0.0015 (6)	0.0074 (6)	0.0004 (6)
C10	0.0233 (8)	0.0164 (8)	0.0237 (8)	0.0013 (6)	0.0053 (6)	−0.0014 (6)
C11	0.0321 (9)	0.0232 (9)	0.0199 (8)	0.0047 (7)	0.0040 (7)	0.0001 (7)
C12	0.0362 (9)	0.0215 (8)	0.0229 (8)	0.0053 (7)	0.0127 (7)	0.0044 (7)
C13	0.0254 (8)	0.0220 (8)	0.0283 (9)	0.0011 (6)	0.0117 (7)	0.0035 (7)
C14	0.0226 (8)	0.0150 (7)	0.0206 (8)	0.0006 (6)	0.0073 (6)	−0.0009 (6)
C15	0.0221 (8)	0.0312 (9)	0.0215 (8)	0.0024 (7)	0.0011 (7)	0.0047 (7)
C16	0.0202 (8)	0.0394 (11)	0.0284 (9)	0.0058 (7)	0.0084 (7)	0.0089 (8)
C17	0.0294 (9)	0.0230 (8)	0.0222 (8)	0.0026 (7)	0.0106 (7)	0.0007 (7)
C18	0.0319 (9)	0.0216 (8)	0.0288 (9)	0.0050 (7)	0.0146 (8)	0.0047 (7)
C19	0.0305 (9)	0.0251 (9)	0.0203 (8)	−0.0082 (7)	0.0036 (7)	−0.0004 (7)
C20	0.0396 (10)	0.0300 (10)	0.0173 (8)	−0.0114 (8)	0.0028 (7)	−0.0022 (7)

### Geometric parameters (Å, °)

**Table:**

O1—C7	1.315 (2)	C6—H6	0.9500
O1—H1O	0.8402	C8—C13	1.411 (2)
O2—C7	1.223 (2)	C8—C9	1.419 (2)
O3—C14	1.290 (2)	C9—C10	1.397 (2)
O4—C14	1.243 (2)	C9—C14	1.507 (2)
N1—C1	1.384 (2)	C10—C11	1.384 (2)
N1—H1N	0.8800	C10—H10	0.9500
N1—H2N	0.8802	C11—C12	1.394 (3)
N2—C8	1.379 (2)	C11—H11	0.9500
N2—H3N	0.8800	C12—C13	1.382 (3)
N2—H4N	0.8799	C12—H12	0.9500
N3—C17	1.479 (2)	C13—H13	0.9500
N3—C19	1.480 (2)	C15—C16	1.540 (2)
N3—C15	1.485 (2)	C15—H15A	0.9900
N4—C18	1.483 (2)	C15—H15B	0.9900
N4—C20	1.488 (2)	C16—H16A	0.9900
N4—C16	1.489 (2)	C16—H16B	0.9900
N4—H5N	0.9300	C17—C18	1.536 (2)
C1—C6	1.411 (2)	C17—H17A	0.9900
C1—C2	1.417 (2)	C17—H17B	0.9900
C2—C3	1.397 (2)	C18—H18A	0.9900
C2—C7	1.494 (2)	C18—H18B	0.9900
C3—C4	1.388 (2)	C19—C20	1.541 (2)
C3—H3	0.9500	C19—H19A	0.9900
C4—C5	1.395 (2)	C19—H19B	0.9900
C4—H4	0.9500	C20—H20A	0.9900
C5—C6	1.384 (2)	C20—H20B	0.9900
C5—H5	0.9500		
			
C7—O1—H1O	108.5	C10—C11—H11	120.6
C1—N1—H1N	114.1	C12—C11—H11	120.6
C1—N1—H2N	113.2	C13—C12—C11	120.57 (16)
H1N—N1—H2N	119.4	C13—C12—H12	119.7
C8—N2—H3N	118.3	C11—C12—H12	119.7
C8—N2—H4N	119.5	C12—C13—C8	121.35 (16)
H3N—N2—H4N	119.5	C12—C13—H13	119.3
C17—N3—C19	109.10 (14)	C8—C13—H13	119.3
C17—N3—C15	108.67 (13)	O4—C14—O3	123.47 (15)
C19—N3—C15	109.31 (14)	O4—C14—C9	120.27 (15)
C18—N4—C20	109.67 (14)	O3—C14—C9	116.19 (14)
C18—N4—C16	109.51 (14)	N3—C15—C16	109.79 (13)
C20—N4—C16	109.83 (14)	N3—C15—H15A	109.7
C18—N4—H5N	109.3	C16—C15—H15A	109.7
C20—N4—H5N	109.3	N3—C15—H15B	109.7
C16—N4—H5N	109.3	C16—C15—H15B	109.7
N1—C1—C6	118.89 (15)	H15A—C15—H15B	108.2
N1—C1—C2	122.85 (14)	N4—C16—C15	109.08 (13)
C6—C1—C2	118.18 (15)	N4—C16—H16A	109.9
C3—C2—C1	119.56 (14)	C15—C16—H16A	109.9
C3—C2—C7	119.14 (14)	N4—C16—H16B	109.9
C1—C2—C7	121.29 (14)	C15—C16—H16B	109.9
C4—C3—C2	121.70 (15)	H16A—C16—H16B	108.3
C4—C3—H3	119.2	N3—C17—C18	110.71 (13)
C2—C3—H3	119.2	N3—C17—H17A	109.5
C3—C4—C5	118.66 (16)	C18—C17—H17A	109.5
C3—C4—H4	120.7	N3—C17—H17B	109.5
C5—C4—H4	120.7	C18—C17—H17B	109.5
C6—C5—C4	120.95 (15)	H17A—C17—H17B	108.1
C6—C5—H5	119.5	N4—C18—C17	108.48 (13)
C4—C5—H5	119.5	N4—C18—H18A	110.0
C5—C6—C1	120.87 (15)	C17—C18—H18A	110.0
C5—C6—H6	119.6	N4—C18—H18B	110.0
C1—C6—H6	119.6	C17—C18—H18B	110.0
O2—C7—O1	123.04 (15)	H18A—C18—H18B	108.4
O2—C7—C2	123.56 (15)	N3—C19—C20	110.12 (14)
O1—C7—C2	113.39 (14)	N3—C19—H19A	109.6
N2—C8—C13	119.39 (16)	C20—C19—H19A	109.6
N2—C8—C9	122.57 (15)	N3—C19—H19B	109.6
C13—C8—C9	118.03 (15)	C20—C19—H19B	109.6
C10—C9—C8	119.14 (15)	H19A—C19—H19B	108.2
C10—C9—C14	117.83 (15)	N4—C20—C19	108.78 (14)
C8—C9—C14	122.98 (15)	N4—C20—H20A	109.9
C11—C10—C9	122.13 (16)	C19—C20—H20A	109.9
C11—C10—H10	118.9	N4—C20—H20B	109.9
C9—C10—H10	118.9	C19—C20—H20B	109.9
C10—C11—C12	118.74 (16)	H20A—C20—H20B	108.3
			
N1—C1—C2—C3	−179.44 (14)	C11—C12—C13—C8	1.6 (3)
C6—C1—C2—C3	−2.8 (2)	N2—C8—C13—C12	179.51 (17)
N1—C1—C2—C7	1.4 (2)	C9—C8—C13—C12	−1.6 (2)
C6—C1—C2—C7	178.02 (14)	C10—C9—C14—O4	−19.9 (2)
C1—C2—C3—C4	1.9 (2)	C8—C9—C14—O4	162.91 (15)
C7—C2—C3—C4	−178.95 (15)	C10—C9—C14—O3	157.33 (15)
C2—C3—C4—C5	0.6 (2)	C8—C9—C14—O3	−19.9 (2)
C3—C4—C5—C6	−2.2 (3)	C17—N3—C15—C16	−62.35 (18)
C4—C5—C6—C1	1.2 (3)	C19—N3—C15—C16	56.62 (19)
N1—C1—C6—C5	178.09 (15)	C18—N4—C16—C15	57.55 (19)
C2—C1—C6—C5	1.3 (2)	C20—N4—C16—C15	−62.94 (19)
C3—C2—C7—O2	−171.35 (16)	N3—C15—C16—N4	5.0 (2)
C1—C2—C7—O2	7.8 (2)	C19—N3—C17—C18	−62.15 (18)
C3—C2—C7—O1	9.0 (2)	C15—N3—C17—C18	56.95 (18)
C1—C2—C7—O1	−171.82 (15)	C20—N4—C18—C17	57.54 (18)
N2—C8—C9—C10	178.78 (16)	C16—N4—C18—C17	−63.04 (18)
C13—C8—C9—C10	−0.1 (2)	N3—C17—C18—N4	4.8 (2)
N2—C8—C9—C14	−4.0 (3)	C17—N3—C19—C20	55.90 (18)
C13—C8—C9—C14	177.12 (15)	C15—N3—C19—C20	−62.81 (18)
C8—C9—C10—C11	1.8 (2)	C18—N4—C20—C19	−63.53 (19)
C14—C9—C10—C11	−175.57 (15)	C16—N4—C20—C19	56.86 (19)
C9—C10—C11—C12	−1.8 (3)	N3—C19—C20—N4	5.3 (2)
C10—C11—C12—C13	0.0 (3)		

### Hydrogen-bond geometry (Å, °)

**Table:**

*D*—H···*A*	*D*—H	H···*A*	*D*···*A*	*D*—H···*A*
O1—H1O···N3	0.84	1.77	2.597 (2)	168
N4—H5n···O3	0.93	1.64	2.546 (2)	166
N1—H2n···O2	0.88	2.03	2.725 (2)	135
N2—H3n···O3	0.88	2.04	2.696 (2)	131
N1—H1n···O4^i^	0.88	2.08	2.941 (2)	165
N2—H4n···N1^ii^	0.88	2.38	3.256 (2)	171

Symmetry codes: (i) −*x*+1, *y*−1/2, −*z*+3/2; (ii) −*x*+2, *y*+1/2, −*z*+3/2.
